# A comparison of patients with relapsed and chemo-refractory gestational trophoblastic neoplasia

**DOI:** 10.1038/sj.bjc.6603608

**Published:** 2007-02-13

**Authors:** T Powles, P M Savage, J Stebbing, D Short, A Young, M Bower, C Pappin, P Schmid, M J Seckl

**Affiliations:** 1 Department of Medical Oncology, Charing Cross Gestational Trophoblastic Disease Centre, Hammersmith Hospitals Campus of Imperial College London, Fulham Palace Road, London, W6 8RF, UK

**Keywords:** gestational trophoblastic neoplasia, relapse, chemotherapy

## Abstract

The majority of women requiring chemotherapy for gestational trophoblastic disease (GTN) are cured with their initial chemotherapy treatment. However, a small percentage either become refractory to treatment, or relapse after the completion of treatment. This study investigates the characteristics and outcome of these patients. Patients were identified from the Charing Cross Hospital GTD database. The outcome of these patients with relapsed disease was compared to those with refractory disease. Between 1980 and 2004, 1708 patients were treated with chemotherapy for GTN. Sixty (3.5%) patents relapsed following completion of initial therapy. The overall 5-year survival for patients with relapsed GTN was 93% (95% CI 86–100%). The overall survival for patients with low-risk and high-risk disease at presentation, who subsequently relapsed was 100% (*n*=35), and 84% (*n*=25) (95% CI: 66–96%: *P*<0.05), respectively. Eleven patients were identified who failed to enter remission and had refractory disease. These patients had a worse outcome compared to patients with relapsed disease (5-year survival 43% (95% CI:12–73% *P*<0.01)). The outcome of patients with relapsed GTN is good. However, patients with primary chemo-refractory disease do poorly and novel therapies are required for this group of patients.

Gestational trophoblastic disease comprises a spectrum of disorders ranging from the premalignant complete and partial hydatidiform moles to the malignant invasive mole, choriocarcinoma and rare placental site trophoblastic tumours (PSTT). The latter three conditions are also collectively known as gestational trophoblastic tumours or neoplasia (GTN). The majority of patients with GTN are cured with chemotherapy. The intensity of the initial chemotherapy for invasive mole and choriocarcinoma is dependant on the risk factors at presentation, with patients stratified into low- and high-risk prognostic groups (Ngan, 2004). These two groups of patients are treated differently. The low-risk patients receive single-agent therapy, most commonly with methotrexate, whereas high-risk patients are treated with combination chemotherapy, using weekly combination chemotherapy, comprising of etoposide, methotrexate, actinomycin alternating with cyclophosphamide and vincristine (EMA-CO) at our institution (Bagshawe, 1976). The outcome of both the groups of patients is good with 5-year survival of 100% for low-risk and 86% for high-risk patients (Farhat *et al*, 1996; Bower *et al*, 1997). However, a small minority of patients with GTN will relapse after the completion of treatment.

Owing to the rarity of the disease and the small number of patients relapsing after treatment, there are few publications examining the management and outcome of this group. Additionally, previous studies have combined relapsed patients with those that have progressive disease on chemotherapy (refractory disease) (Lurain and Nejad, 2005; Matsui *et al*, 2005). However, in other curable cancers such as lymphoma and germ cell tumours, there is evidence that the outcome for relapsed as opposed to primary refractory disease is different (Proctor *et al*, 2001; McNeish *et al*, 2004). Here, we separately describe and compare the characteristics, management and outcome of patients who relapse following chemotherapy with those who have primary chemotherapy refractory disease.

## PATIENTS AND METHODS

The Charing Cross GTD database includes full clinical details of women diagnosed with GTN. It was screened to identify women who had primary refractory disease or relapsed after completion of chemotherapy for GTN between 1980 and 2004.

### Patients with relapsed disease

Relapse was defined as two elevated and increasing serum human chorionic gonadotropin (hCG) levels, in the absence of a normal pregnancy, after achieving complete serological remission with chemotherapy. Complete remission was defined as a minimum of 6 weeks of normal hCG values on chemotherapy. At this point, chemotherapy was stopped and patients entered the post-treatment screening programme. The post-treatment screening programme involves once weekly blood tests for 6 weeks, then two weekly blood and urine samples up to 6 months, followed by urine samples two weekly for the rest of the first year. From there, urine hCG is measured at increasing intervals up to 5 years. At this point urine is requested every 6 months for life.

### Patients with refractory disease to EMA/CO therapy

This group includes high-risk GTN patients whose hCG had increased, or failed to decrease (plateau) on EMA/CO chemotherapy. Human chorionic gonadotropin levels were assessed twice weekly on treatment according to the protocol. As hCG can initially rise and/or take 2–4 weeks before falling following commencement of chemotherapy, patients were given at least 3 weeks of EMA/CO before a diagnosis of refractory disease could be made.

Refractory disease was defined as:

1. Two or more abnormal and increasing plasma hCG levels, despite receiving standard EMA/CO chemotherapy for at least 3 weeks.
2. Three or more consecutive hCG values that had failed to fall more than 5% below the preceding hCG level. These patients were considered to have a plateau in their hCG.

Patients with refractory disease went on to receive platinum-based chemotherapy (see later for treatment details). Nine patients with high-risk disease were excluded from this study because they died early from GTN, before a fall in the hCG could be attributed to chemotherapy resistance.

### Other exclusion criteria

Patients with PSTT were excluded from this analysis because the treatment and outcomes for these patients are different (Papadopoulos *et al*, 2002)

### Details at initial diagnosis of GTN

At initial diagnosis, patients were staged according to the national and internationally accepted criteria for GTN, and then stratified into low- or high-risk groups and treated accordingly (Bagshawe, 1976; Kohorn, 2001). Patients were treated in a uniform manner using the standard well-established treatment protocols of methotrexate/folinic acid and EMA-CO. Women with in the low-risk group received intramuscular methotrexate with folinic acid. In the event of resistance to methotrexate, characterised by a rise or plateau in the serum hCG, treatment was switched to actinomycin D or the EMA-CO high-risk treatment regimen, depending on the hCG level as described previously (McNeish *et al*, 2002). The majority of patients with high-risk disease were commenced EMA/CO. However, in the presence of brain or liver metastasis different regimens were used (EMA/CNS (high doses of methotrexate) or EP/EMA (EP=etoposide and cisplatin), respectively) (Rustin *et al*, 1989; Bower *et al*, 1997; Newlands *et al*, 2000).

### Investigations and treatment at diagnosis of relapse of GTN

Patients who relapsed underwent restaging, which included a pelvic Doppler US, CT body and CT or MRI brain. The treatment for patients who relapsed after methotrexate or actinomycin for (low-risk disease) was either surgery alone (if the relapse was isolated) or EMA/CO with or without surgery. For those relapsing after EMA/CO (high-risk disease) treatment was either surgery and/or EP/EMA. Relapsed patients were stratified into relapse after initial high- and low-risk treatment for comparative purposes.

### Investigations and treatment at diagnosis of refractory disease

The details and characteristics of these patients were noted at the time of development of refractory disease. Patients went on to receive platinum-based chemotherapy usually EP/EMA (Newlands *et al*, 2000).

### Statistical methods

Survival was calculated from the day of diagnosis until death or the date of last follow-up. Overall survival duration curves were plotted according to the method of Kaplan and Meier. Comparison of variables between groups was by *χ*^2^ test for nominal variables, and Mann–Whitney *U*-test for non-parametric variables, log-rank tests were used to compare overall survival over time and all *P*-values presented are two-sided. Univariate analysis was performed on the cohort with relapsed disease.

## RESULTS

### Patients characteristics (Table 1 and Figure 1)

From a database of 1708-treated GTN patients, 60 (3.5%) relapsed after chemotherapy, 35 of 1397 (2%) low-risk patients relapsed compared to 25 of 311 (8%) high-risk patients. Eleven patients fulfilled the criteria for disease refractory to EMA/CO chemotherapy.

### Details of relapsed patients at initial presentation of GTN (Table 1)

The characteristics of the 60 patients who relapsed are given in Table 1. At initial presentation, 35 (58%) patients were in the low-risk prognostic group and received single-agent methotrexate, eight of these patients became resistant to methotrexate and changed to more intensive chemotherapy two with EMA/CO and six with actinomycin (McNeish *et al*, 2000).

The remaining 25 (42%) patients initially presented with high-risk disease and were treated accordingly: 17 received EMA/CO chemotherapy, six patients received platinum containing regimens or other regimens including cisplatin etoposide (*n*=1), cisplatin etoposide and vincristine (*n*=1). These other regimens were used because of the presence of extra-pulmonary metastasis, intolerance of methotrexate or treatment predating the routine introduction of EMA/CO chemotherapy.

### Details of patients at relapse (Table 2)

The characteristics of the patients at the time of relapse are shown in Table 2. The majority of relapses (73%) occurred within 1 year of completing initial chemotherapy and only one patient relapsed after more than 5 years of follow-up (Table 3). Human chorionic gonadotropin screening of either blood or urine samples identified 47 (78%) of these relapses. Thirteen represented with symptoms (predominantly amenorrhoea), including two patients who did not comply with screening and presented with symptomatic relapse.

### Treatment at relapse of GTN (Table 4)

Treatment at the time of relapse depended on a number of factors. The most important of which were previous treatment at initial presentation (high- *vs* low-risk prognostic group) and site of disease.

Most women initially treated with methotrexate for low-risk disease received EMA/CO at relapse, although other regimens were used in five patients due to intolerance of EMA/CO (Table 4). Relapse after initial high-risk chemotherapy was treated with platinum-based chemotherapy in the majority of patients, most frequently the EP/EMA regimen (15 out of 25). EMA/CO was reused successfully at relapse in three patients with a long treatment-free interval (>1 year). Patients with brain metastasis received EMA/CO with high-dose methotrexate (Rustin *et al*, 1989).

Surgery was performed, in conjunction with chemotherapy, in 15 women with easily resectable metastasis isolated from one organ. Two patients with low-risk disease and isolated uterine relapse underwent a hysterectomy without chemotherapy owing to patient preference, one of these patients relapsed and required chemotherapy and was cured.

There were no treatment-related deaths in this group of patients with relapsed GTN.

### Outcome of patients with relapsed GTN (Table 4)

After a median follow-up of 10.8 years (range 1.7–24.5) after relapse, there have been five deaths among the 60 patients with relapsed disease, all owing to progressive chemotherapy-resistant GTN. These patients received third-line therapy (high-dose treatment in four (McNeish *et al*, 2004)). Although markers temporarily normalised in two of these patients, none went into a sustained complete remission. Ultimately, all patients progressed and died of progression of disease in multiple sites. All five deaths occurred in patients falling into the initial high-risk prognostic group; none of these patients who died achieved a complete remission with second-line platinum-based chemotherapy. All of these five patients progressed on third-line therapy. Indeed all of the women who went into complete remission after second-line treatment are alive, whereas all patients who progressed on second-line treatment have died of GTN. The 5-year overall survival was 93% (95% CI: 68–100%), this dropped to 84% (95%CI: 68–99%) for patient with high-risk disease. None of the patients initially within the low-risk prognostic group died.

Overall nine patients have relapsed for a second time; three from the initial low-risk disease group and six from the initial high-risk group. The median time interval between first and second relapse was 0.8 years (range 0.3–4.6). All of these patients went into complete remission with third-line treatment and are alive with a median follow-up of 13.0 years (range 3.0–22.3). This third-line treatment included surgery (*n*=1), chemotherapy (*n*=5) and chemotherapy and surgery (*n*=3). The chemotherapy regimens used included high-dose therapy (*n*=2) and, EP/EMA (*n*=5), EMA/CO (*n*=1). Indeed two of these patients have relapsed for a third time and are in remission with fourth-line chemotherapy, with 4.5 and 7.3 years of follow-up.

### Prognostic factors for patients with relapsed disease (Table 5)

A number of clinical variables were found to be of prognostic importance using univariate analysis. These included the presence of metastasis at relapse, where the 5-year overall survival was 79% (95%CI: 61–97) for patients with metastasis and 97% (95%CI: 87–100) for patients without metastasis. Other significant factors included a slow hCG-doubling time at relapse, high-risk disease at presentation and term pregnancies. Multivariate analysis was precluded due to the small sample size in conjunction with the small number of events.

### Patients with refractory disease (Table 1 and Figure 2)

Eleven patients progressed on EMA/CO chemotherapy and fulfilled the criteria for refractory disease. Seven of these patients had consecutive rising hCG levels on chemotherapy, whereas the other four had a plateau in their hCG. All of these patients received platinum-based chemotherapy (EP/EMA 8, EP 3). Six of these patients have died with a median overall survival of 1.2 years. All six patients died of chemoresistant GTN, four of these patients subsequently received high-dose therapy. These patients have a poor prognosis compared to those with relapsed disease after high-risk treatment (5-year survival 43% (95% CI: 68–99%) *vs* 84% (95%CI: 12–73% (*P*=0.003)). It is noteworthy that these patients had poor prognostic at diagnosis features compared to the high-risk relapse patients (Table 1).

## DISCUSSION

This is the largest reported series of GTN patients with either relapsed disease after chemotherapy or refractory disease. The main findings are that the relapse rate following chemotherapy is low (3.5%) and the majority of these patients can be cured with further treatment (>90%). However, patients with disease which is refractory to high-risk treatment have a significantly less good outcome.

None of the women who relapsed after low-risk disease died, which is reassuring for these patients. Additionally, extrapolating from previously published data, the 5-year survival of patients with relapse after high-risk disease is similar to that for high-risk patients at initial presentation (84% (95% CI: 68–99%) *vs* 86% (95%CI: 82–91%), respectively) (Bower *et al*, 1997; Dobson *et al*, 2000). All of the relapsed patients who died failed to go into complete remission with second-line chemotherapy at relapse. Therefore, obtaining a complete serological remission with second-line chemotherapy is crucial for these patients.

This work also shows the outlook for patients with chemotherapy refractory disease is less good. These findings are consistent with data from other tumour types such as ovary and testis, which demonstrate a difference in outcome regarding patients with refractory and relapsed disease (Thigpen *et al*, 1993; McCaffrey *et al*, 1997; Herzog, 2002).

Owing to the lack of published data, there is no specific scoring/prognostic system for relapsed or refractory patients. The WHO and FIGO scoring systems are used to separate women into high- and low-risk disease at initial presentation. Although these scoring systems do include prognostic scores (points) for previous treatment (up to six points), the scoring system has not been widely evaluated or validated for these rare patients. Although numbers are small, the data presented here do highlight a number of factors which were of poor prognostic importance in univariate analysis, these include metastatic disease at relapse, previous high-risk treatment, previous nonpulmonary metastases, nonmolar pregnancies and a slow hCG-doubling time (>10 days). These factors may give us a better insight into predicting the outcome of these patients. For example, none of the women with two or less of these factors have died. Unfortunately, in view of the rarity of the disease and small number of deaths in this cohort, it is not possible to construct and validate a prognostic scoring system.

The observation that patients with a slow hCG-doubling time at relapse appear to have a worse outcome compared to those with a rapid doubling time is interesting. The reasons for this are unclear, but a slow doubling time may be a mark of a slow growing, chemotherapy-resistant tumour or a tumour that no longer secretes hCG appropriately due to mutation or differentiation as has been described with other cancers (Fossa *et al*, 1992). However, the time between initial diagnosis and relapse was not significant in univariate analysis. This may in part be due to the small numbers because of the rarity of the disease.

Currently, clinicians at the Charing Cross Hospital are treating patients with relapse after methotrexate for low-risk disease with EMA/CO, which seems appropriate as the overall survival is 100% in this group. EMA/CO is first-line high-risk treatment at the Charing Cross; however, other regimens such as MEA (methotrexate, etoposide and actinomycin) (Dobson *et al*, 2000) are used at other centres. These appear to have similar relapse and overall survival rates, although direct comparison as part of a study has not been performed. The majority of patients who relapsed after initial high-risk treatment in this series received EP/EMA, which has previously been shown to be a potent regimen in GTN (Newlands *et al*, 2000). Three high-risk relapse patients were retreated with EMA/CO because of a long interval between treatments (>1 year). None of these three have relapsed, which gives us some insight into the mechanism of chemotherapy resistance in this tumour type. Paclitaxel in combination with cisplatin and etoposide is a promising regimen in chemotherapy-resistant GTN and used successfully twice in this series (Osborne *et al*, 2004).

The role of surgery in the management of relapse GTN is unknown. It is theoretically curative without the need for further chemotherapy, if complete excision is achieved and postoperatively the hCG falls with an appropriate half-life of 1–2 days. However, this study shows that surgery alone may be inadequate, and so the decision not to give further chemotherapy should be carefully considered. If the disease is isolated in the uterus, hysterectomy plus chemotherapy is a safe option and may reduce the duration of chemotherapy treatment (Soper, 2003). However, the majority of women maintain their fertility after chemotherapy and therefore, if a patient wishes to have children, chemotherapy alone is preferable (Bower *et al*, 1997).

Owing to the sensitive and specific nature of hCG in GTN, screening has been offered to relapsed patients after treatment. The data in this study show that most patients relapse within the first 6 months and the majority are detected by the screening programme. This finding confirms the benefit of the testing schedule for these women following chemotherapy. It is interesting that 15% patients relapsed after 2 years, which underlines the need to continue monitoring these women.

This study also investigated patients with disease refractory to high-risk therapy. These patients have poor outcome compared to those with relapsed disease and once again underlines the importance of obtaining a normal hCG with treatment for GTN.

There are two other publications in this area, both of which combined patients with resistant and relapsed disease together (Lurain and Nejad, 2005, Matsui *et al*, 2005). In the study by Matsui *et al*, only six patients with low-risk disease relapsed, and therefore, it is difficult to draw too many conclusions from this, although none have died, which is consistent with our data. The other study focused on 26 patients with high-risk disease and did not distinguish between refractory disease and relapse. As we have shown in this study, these two groups of patients have differing outcomes, making the interpretation of their results complex.

Although all patients in this series were initially diagnosed with histologically confirmed gestational trophoblastic disease, it is possible that some of the high-risk patients who were refractory or relapsed may in fact have had non-gestational hCG producing tumours that masquerade as GTN (Fisher and Newlands, 1998). In a number of isolated cases, we have performed genetic analysis to confirm the non-gestational genetic origin of the tumour. However, it is not possible to perform testing in all cases. Therefore, it is conceivable that there will be a small proportion of non-gestational tumours in this series. An additional shortcoming of this work is that none of the relapsed tumours were genetically tested to confirm that the disease was truly relapse, rather than a second unrelated tumour. However, there are a number of factors, which strongly support the theory that these patients have relapsed. These include the short period between completing chemotherapy and relapse of disease (median 4 months). Additionally, the patients with relapsed disease presented in a different manner to those at initial presentation, with a higher proportion with metastatic disease, comparatively low hCGs and low burden of disease in the uterous. Indeed no abnormality was noted in the uterous in 41% of patients at relapse. Despite this, it is not possible to completely rule out the possibility that some of these tumours were due to a second primary tumour without genetic testing.

In summary, the outcome of women with relapsed GTN is good, especially those initially treated with low-risk disease. The outcome for patients with refractory disease is less favourable. Monitoring patients after relapse is recommended for at least 5 years

## Figures and Tables

**Figure 1:**
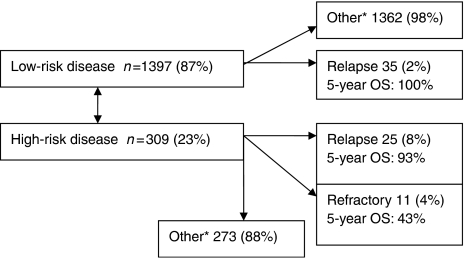
Origin of patients with relapsed or refractory GTN.

**Figure 2:**
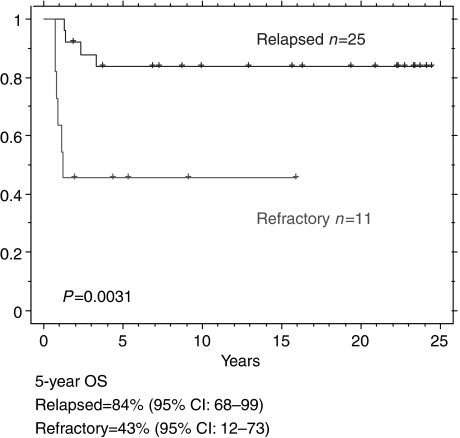
Outcome of patients who relapsed after high-risk treatment *vs* refractory (progression on initial high-risk chemotherapy).

**Table 1:** Characteristics at initial presentation of disease for patients who developed relapsed GTN (*n*=60)

**Characteristic**	**All relapsed patients (high- and low- risk at initial diagnosis)**	**Patients with high-risk disease at initial diagnosis who relapse**	**Refractory disease**
Number	60	25	11
Median prognostic score (WHO) at diagnosis	7 (range 2–23)	9 (range 8–23)	16 (range 8–26)
Median prognostic score (FIGO)	4 (range 1–12)	7 (range 5–12)	10 (range 6–18)
Antecedent pregnancy	Term 19 Abortion 7 Mole 34	Term 17 Abortion 5 Mole 3	Term 7 Abortion 1 Mole 3
Median hCG before initial treatment	21 000 (range 31–814 000)	66 000 (range 350–814 000)	380 000 (range 79 000–1 100 000)
Duration between causal pregnancy and initial treatment	0.4 years (range 0.1–7.3 years)	1.1 years (range 0.1–7.3 years)	3.2 years (range 0.1–15)
Uterine size	0–3 cm–15 3–5 cm–39 >5 cm–6	0–3 cm–7 3–5 cm–16 >5 cm–2	0–3 cm5 3–5 cm–4 >5 cm–2
Site of metastasis	None 40 Lung only 16 Other sites^a^ 4	None 12 Lung only 9 Other sites^a^ 4	None 0 Lung only 7 Other sites^b^ 4
Initial treatment regimens	Methotrexate 35 EMA/CO 18 Platinum-based 7	EMA/CO 17 Platinum-based 7	EMA/CO 11

GTN=gestational trophoblastic neoplasia; WHO=World Health Organization.

a With or without lung metastasis (includes liver (2), brain (1) and liver and brain(1).

b Liver, 2; brain, 2.

**Table 2:** Patients characteristics at time of relapse or refractory disease

	**Patients with relapsed disease**	**Patients with refractory disease**
Number	60	11
Median time to relapse or refractory disease (months)	4 months (range 0.25–37 months)	3 months (range 0.25–6.75)
Median hCG at relapse/refractory disease	309 (range 25–105 000)	1244 (range 14–255 000)
Rate of hCG rise (median doubling time in days)	9 days (range 3–28) days)	NA
Median time between diagnosis of relapse and starting treatment (months)	0.4 months (range 0–3)	NA
Symptoms/method of diagnosis of relapse	hCG Screening 47 PV bleeding 2 Amenorrhoea 7 Other^a^ 4	Screening 11
Site of disease at relapse	hCG relapse12 Mass in uterus only 29 Lung 12 Lung and uterus 3 Pelvis 1 other and uterus 3^b^ Unknown 1	NA

HCG=human chorionic gonadotropin; NA=not available.

a Respiratory symptoms, haemoptysis and re-entry to screening programme.

b Includes liver (1), liver and brain(1), pelvis (2).

**Table 3:** Time to relapse after chemotherapy

**Time to relapse**	**Number (%)**	**Cumulative (%)**
0–3 months	31 (51.6)	51.6
3–6 months	10 (16.6)	68.2
6–12 months	3 (5)	73.2
12–24 months	7 (11.7)	84.9
1–5 years	8 (13.5)	98.4
>5 years	1 (1.6)	100

**Table 4:** Management and outcome of relapsed disease

*Low risk at initial presentation patients n=35*	
Hysterectomy only	2
Hysterectomy and chemotherapy	EMA/CO 3 EP 1
Chemotherapy only	EMA/CO 25 CO/EP 1 EP 1 EP/EMA 2
5-year survival	100%
	
*High risk at initial presentation patients n=25*	
Thoracotomy and chemotherapy	EP/EMA 2 POMB ACE 1 EMA/CO 1
Hysterectomy and chemotherapy	EP/EMA 3 EMA/CO 1 Cis/taxol 1
Chemotherapy only	EMA/CNS 1 EMA/CO 3 EP/EMA 10 Cisplatin vincristine 1 Cis/taxol 1
5-year survival	84% (95% CI: 68–99%)

EMA/CO – weekly combination chemotherapy, comprising of etoposide(dose), methotrexate, actinomycin alternating with cyclophosphamide and vincristine (Bower *et al*, 1997).

POMBACE – cisplatin, vincristine, methotrexate, bleomycin alternating with actinomycin D, cyclophosphamide and etoposide (Newlands *et al*, 1983).

EP/EMA – etoposide, cisplatin/etoposide, methotrexate and actinomycin D (Newlands *et al*, 2000).

EMA/CNS – etoposide, methotrexate and actinomycin alternating weekly with vincristine and cyclophosphamide. The dose of methotrexate was increased to 1 g m^−2^.

Cis/taxol – cisplatin etoposide and paclitaxel (Osborne *et al*, 2004) EP cisplatin etoposide (IV day 1–5).

**Table 5:** Univariate analysis for patients with relapsed disease

		**GTN-related deaths**		
		**Yes**	**No**	***P*-value**
Median age at relapse (years)		32	29	0.26
Median hCG at presentation		15 000	28 000	0.14
Median interval between causal pregnancy and first chemotherapy		1.1 years	0.5 years	0.57
Maximum hCG at relapse (medium)		350	121	0.41
HCG doubling time at relapse (days)		15	7	0.01^*^
Lung metastasis at presentation	No	2	42	0.11
	Yes	3	13	
Nonpulmonary metastasis at presentation	No	3	52	0.05^*^
	Yes	2	3	
Treatment at initial presentation	High risk	5	20	0.01^*^
	Low risk	0	35	
Interval between chemotherapy and relapse	0–3 months	4	30	0.19
	>3 months	1	25	
Metastasis identified at relapse	No	1	39	0.03^*^
	Yes	4	16	
Uterine mass identified at relapse	No	3	24	0.65
	Yes	2	31	
Nonpulmonary metastasis at relapse	No	4	52	0.30
	Yes	1	3	
Causal pregnancy	Mole	0	29	0.02^*^
	Term	5	26	
